# Pre-emergence herbicides used in urban and agricultural settings: dissipation and ecological implications

**DOI:** 10.1007/s10653-024-02269-9

**Published:** 2024-11-07

**Authors:** Aney Parven, Islam Md Meftaul, Kadiyala Venkateswarlu, Mallavarapu Megharaj

**Affiliations:** 1 Global Centre for Environmental Remediation (GCER), School of Environmental and Life Sciences, College of Engineering, Science and Environment, The University of Newcastle, ATC Building, University Drive, Callaghan, NSW 2308 Australia; 2 Department of Agricultural Chemistry, Sher-e-Bangla Agricultural University, Dhaka, 1207 Bangladesh; 3 Formerly Department of Microbiology, Sri Krishnadevaraya University, Anantapuramu, 515003 India; 4 Cooperative Research Centre for Contamination Assessment and Remediation of the Environment (CRC CARE), The University of Newcastle, Callaghan, NSW 2308 Australia

**Keywords:** Pre-emergence herbicides, Dissipation, Soil health, Environmental hazard, Nontarget effects

## Abstract

Herbicides are widely recognized as the most cost-effective solution for weed control, but their extensive use in both urban and agricultural settings raise serious concerns about nontarget effects. We assessed the possible hazards associated with pre-emergence herbicides such as dimethenamid–P, metazachlor, and pyroxasulfone, which are frequently applied in both urban and agricultural soils. The dissipation rate constant values (*k* day^−1^: 0.010–0.024) were positively linked to total organic carbon (TOC), silt, clay, soil pH, and Al and Fe oxides, but negatively correlated with sand content. In contrast, half-life values (DT_50_: 29–69 days) of the herbicides showed negative correlations with TOC, clay, silt, soil pH, and Fe and Al oxides, while sand content showed a positive impact. The selected herbicides showed minimal impact on soil dehydrogenase activity (DHA). Mostly, soils with higher organic matter (OM) content exhibited increased DHA levels, highlighting the role of OM in influencing this soil enzyme across different soils. Assessment of environmental indicators like groundwater ubiquity score (GUS:1.69–6.30) and leachability index (LIX: 0.23–0.97) suggested that the herbicides might reach groundwater, posing potential risks to nontarget biota and food safety. Human non-cancer risk evaluation, in terms of hazard quotient (HQ < 1) and hazard index (HI < 1), suggests minimal or no risks from exposure to soil containing herbicide residues at 50% of the initial concentrations. Our data thus help the stakeholders and regulatory agencies while applying these pre-emergence herbicides in soils and safeguarding human and environmental health.

**Supplementary Information:**

The online version contains supplementary material available at 10.1007/s10653-024-02269-9.

## Introduction

Herbicides are vital in modern agriculture, empowering farmers to efficiently manage weeds and boost crop production (Bajwa et al., 2015). These chemical compounds are designed to target specific plant species, minimizing damage to cultivated crops (Gage et al., 2019). Nevertheless, the extensive and continuing utilization of herbicides has triggered concerns about their environmental consequences and possible effects on human health (Rani et al., 2021). In contrast, degradation can reduce the herbicide residues levels in soil, as this process break down these chemical compounds into simpler forms over time (Kaur et al., 2021). Soil degradation of herbicides is influenced by various factors, including soil composition, temperature, moisture content, microbial activity, and the specific properties of the herbicide itself (Liu et al., 2020). Herbicides can degrade through physical, chemical, and biological processes, and the rates and pathways of degradation can vary widely depending on these factors. Physical degradation of herbicides in soils typically involves processes such as photolysis and hydrolysis, whereas photolysis occurs when herbicides are exposed to sunlight (Meng et al., 2022). Chemical degradation occurs through chemical reactions with substances present in the soil, such as minerals or organic matter and potentially changing their toxicity and environmental behavior (Liu et al., 2020; Paszko et al., 2016). Conversely, certain bacteria and fungi can utilize herbicides as a carbon and energy source, leading to their decomposition referred to as biological degradation (Bose et al., 2021). However, the effectiveness of this degradation process can vary depending on the availability of suitable microbial populations and environmental conditions.

Some herbicides exhibit slow degradation rates, leading to their persistence in soils for extended periods, result in long-term exposure of nontarget organisms to these chemicals, potentially affecting beneficial soil microorganisms, insects, birds, and other wildlife (Ankit et al., 2020; Noshadi & Homaee, 2018). Not only herbicide but also their degradation products can have unintended effects on nontarget organisms, disrupting ecosystems and leading to imbalances in biodiversity (Dhuldhaj et al., 2023). The loss of certain species or changes in the abundance of key organisms can have far-reaching consequences for ecosystem stability and functioning. Frequent and consistent herbicide use can lead to the development of herbicide-resistant weed species. As certain weeds survive exposure to herbicides, they pass on their resistant traits to subsequent generations, creating populations that are challenging to control (Leon et al., 2021; Travlos et al., 2020). This phenomenon increases the dependence on stronger and potentially more hazardous herbicides, escalating the risk of environmental contamination. One of the most significant concerns is the potential for herbicide residues to leach into groundwater (Dugan et al., 2023). When herbicides are applied to crops or land surfaces, there is a risk of these chemicals being transported through the soil profile and reaching underground water sources. Contaminated groundwater can pose a direct threat to drinking water supplies and have far-reaching consequences for both human and ecosystem health (Zaller, 2020). Also, herbicides that persist in soils can be taken up by plants, entering the food chain may accumulate in plant tissues and subsequently transfer to animals or humans upon consumption (Alengebawy et al., 2021).

Pre-emergence herbicides, such as dimethenamid-P, metazachlor, and pyroxasulfone, that are used both in urban and agricultural settings are effective in weed control. The distinction between urban and agricultural soils lies in their composition, structure, and function. Among them, urban soil attributes undergo significant modifications because of human activities, differing them apart from farming or natural soils (Howard, 2017). Alterations induced in the soil through developing urban infrastructure involve micropore collapse, ped breakdown, increased bulk density, and pollution, leading to increased compaction and reduced fertility (Jim, 2023; Meftaul et al., 2023). In contrast, agricultural soils are managed for optimal crop growth, with practices like tilling and fertilization (Techen et al., 2020). The differences in land use patterns and management practices contribute to variations in nutrient content, microbial diversity, and overall soil health between urban and agricultural environments (Wang et al., 2017). The introduction of nutrients and organic matter (OM) into the soil plays a key role in affecting the structure and activity of microbial populations by enhancing metabolism, subsequently affecting herbicide degradation (Moorman & Keller, 2018). Understanding the extent of degradation and toxicity of such recently used herbicides in both agricultural and urban soils is therefore greatly warranted for sustainable land use planning, as it influences human, environmental and soil health. Acquiring comprehensive knowledge on dissipation of these selected herbicides in agricultural and urban soils becomes imperative for evaluating potential threats to soil, environmental, and human health. Therefore, this study was intended to determine the half-life values (DT_50_, days) of dimethenamid-P, metazachlor, and pyroxasulfone in two urban soils and three agricultural soils, each characterized by varying physicochemical properties, with the objective of assessing human, environmental, and soil health risks.

## Materials and methods

### Soil samples

Two urban soils (Callaghan, CAL; and Fletcher, FLE) and three agricultural soils (Queensland, QLD; Maitland, MAT; and Taree, TAR) were collected from a depth of 0–20 cm from the surface layer. Samples from each soil type were randomly gathered from five locations and mixed thoroughly to create a composite sample. The air-dried samples were sieved using a 2-mm diameter sieve and stored at 21 ± 1 °C for further analysis. The soil texture was determined by the hydrometer method, which measured the percentages of sand, silt, and clay, following the procedure outlined by Gee and Or (2002). Soil pH and electrical conductivity (EC) were measured from 5 g of soil in 25 mL of Milli-Q water. Total organic carbon (TOC) was determined with a LECO analyzer (LECO Corporation, Australia). Functional groups in soil OM were identified using FT-IR spectroscopy (Agilent Technologies, USA). Metal oxides (Al and Fe) were extracted from 0.50 g of soil using a solution of 1HNO_3_ and 3HCl, then digested with Microwave Digestion (MARS 6™, USA). The extracted metals were analyzed using ICP-OES (PerkinElmer Pvt Ltd, Singapore). Soil minerals were detected from peaks found through XRD (PANalytical, The Netherlands). The morphology and superficial elemental composition of soil OM were determined using SEM and EDS at 15 kV after platinum coating (Bruker Nano GmbH, Germany). Elemental mapping using EDS was conducted at 12 keV, employing a step size of 10 μm and an integration time of 0.50 s per step, covering an area of 1.50 × 1.50 mm.

### Chemicals

Standard chemicals of pre-emergence herbicides, namely dimethenamid-P (≥ 98%), metazachlor (≥ 98%), and pyroxasulfone (≥ 95%), along with other reagents like acetonitrile and acetic acid (LC–MS grade), were sourced from Merck. The commercial formulations of these herbicides, such as Outlook (720 g L^‒1^ dimethenamid-P), Butisan (500 g L^‒1^ metazachlor), and Sakura (850 mg g^‒1^ pyroxasulfone), were purchased from CRT Raymond’s Warehouse in Australia. To prepare stock solutions of each herbicide at 100 mg L^‒1^, aliquots of 138.80 μL for dimethenamid-P, 200 μL for metazachlor, and 117.60 μg for pyroxasulfone were taken from the commercial formulations and diluted to 1.0 L with Milli-Q water. Subsequently, 0.80 mL of each 100 mg L^‒1^ herbicide solution was added to 20 g of soil to achieve an ultimate concentration of 4 µg g^‒1^ soil.

### Dissipation experiments

Soil samples, each weighing 20 g, were placed into centrifuge tubes and covered with perforated aluminium foil. These tubes were then stored in darkness at 21 ± 1 °C for 30 days. During incubation, the soil moisture content was adjusted to 15% based on weight. To prepare a final concentration of approximately 4 mg kg^–1^ of the active ingredient in each soil sample, 0.80 mL aliquots of a 100 mg L^–1^ aqueous solution, derived from commercial herbicide formulations, were added to the samples. The soil samples were left for two h after thorough mixing of the herbicide. To maintain the soil moisture at 70% water-holding capacity, required aliquots of Milli-Q water were added. The samples were then incubated in darkness at a constant temperature of 21 ± 1 °C. Meanwhile, a separate set of tubes containing soil and herbicides was stored in a cold room at ‒ 20 °C to evaluate chemical degradation. Control tubes with soil but without any herbicide were also included. At regular intervals (0, 5, 10, 20, 30, 60, 90, 120, and 150 days), duplicate 10-g soil samples were collected for herbicide residue analysis (Hiller et al., 2012; Marin-Benito et al., 2012). The extraction procedure entailed agitating the soil samples with a mixture of 50 mL acetonitrile and water in a 1:1 ratio for three h. Subsequently, the samples were centrifuged at 2750 × *g* for 15 min, and 2-mL portions of the supernatants were used for the analysis of herbicide residues in soil.

### Chemical analysis

The concentrated extracts of selected pre-emergence herbicides were analyzed using liquid chromatography-mass spectrometry (LC–MS) with a Zorbax Eclipse Plus C18 column (4.60 × 150 mm, 3.50 μm dia) from Agilent Technologies, USA. The column temperature was set at 50 °C for metazachlor and dimethenamid-P, and 40 °C for pyroxasulfone. The mobile phases for metazachlor and dimethenamid-P consisted of an aqueous 0.0125% acetic acid solution (A) and acetonitrile (B), with a flow rate of 0.40 mL min^–1^. The gradient commenced at 60% B, increased to 100% B from 6 to 11 min, returned to 30% B at 12 min, followed by a 6-min post-run time (Chen et al., 2018; Lazartigues et al., 2011). For pyroxasulfone, the mobile phases were aqueous 1% acetic acid (A) and acetonitrile (B), with a flow rate of 0.50 mL min^–1^. The gradient commenced at 10% B, increased to 50% B from 15 to 20 min, reached 100% B from 25 to 30 min, and returned to 10% B at 31 min, with a 1-min post-run time. The analysis operated positive mode selective ion monitoring (SIM) for metazachlor and dimethenamid-P, and negative mode for pyroxasulfone. Instrumental parameters included a nebulizer pressure of 35 psi, a drying gas flow of 12 mL min^–1^ at 300 °C, a sheath gas flow of 3 mL min^–1^ at 150 °C, a fragmentor voltage of 100 V, and a capillary voltage of 4000 V. The standard curves for dimethenamid-P, metazachlor, and pyroxasulfone showed linearity with *R*^2^ values of 0.983, 0.993, and 0.995, respectively. The limits of detection (LOD) and quantification (LOQ) for the three herbicides ranged from 0.00097 to 0.00195 mg L^–1^. Mean recoveries (*n* = *3*) for spiked samples ranged from 0.00195 to 1.0 mg L^–1^, with percent recoveries of 81–122% for dimethenamid-P, 83–119% for metazachlor, and 90–113% for pyroxasulfone, demonstrating the method’s accuracy and reliability for herbicide residue analysis in soil. Data processing was conducted using Agilent OpenLAB CDS ChemStation software.

### Determination of half-lives of the herbicides

The rate at which herbicide degrades was measured through the Eq. (1) as follows:

1 $$ lnC_{t} = - kt + lnC_{0} $$

where, C_t_ and C_0_ denote the quantities (mg kg^‒1^) of herbicide present in the soil at time t and initially (at zero time), respectively. The parameter *k* represents the dissipation rate constant (day^‒1^). Subsequently, the DT_50_, indicating the time (days) required for a 50% reduction in the initial herbicide concentration, was derived from *k* using the formula outlined by Hiller et al. (2012):

2 $$ DT_{50} = \frac{ln2}{k} $$

### Assay of soil dehydrogenase activity (DHA)

Soil DHAs were evaluated by determining the conversion of 2,3,5-triphenyltetrazolium chloride (TTC) into triphenylformazan (TPF). Dehydrogenase activities in soil were assessed by measuring the reduction of 2,3,5-triphenyltetrazolium chloride (TTC) to triphenylformazan (TPF). According to the method described by Tabatabai (1994), soil samples weighing 3 g each were mixed with 0.50 mL of a 3% aqueous solution of TTC and 1.25 mL of milli-Q water. After vertexing, the mixtures were left to incubate in darkness at 37 °C for 24 h. Once the incubation period elapsed, each sample received 10 mL of methanol, was mixed well, and then subjected to centrifugation at 2500 × *g* for 20 min. The resulting reddish color intensity was measured using a microplate reader at a wavelength of 485 ηm. DHA was measured and presented as μg of triphenylformazan (TPF) g^–1^ of soil.

### Calculation of environmental health hazards

The groundwater ubiquity score (GUS) and leachability index (LIX) were determined by Eqs. (3) and (4) as outlined earlier (Meftaul et al., 2023).

3 $$ {\text{GUS}} = \log {\text{t}}^{1/2} \left( {4 - \log Koc} \right) $$

4 $$ LIX = \exp \left( { - k \times Koc} \right) $$

5 $$ Koc = \frac{{K_{d} }}{\% OC} \times 100 $$

Equation (3) was used to calculate GUS by including parameters such as the herbicide’s half-life (DT_50_) in soil (days), the degradation rate constant (*k*, in day^−1^), and organic carbon coefficient (*K*_OC_, in L g^−1^) present in the soil. Using Eq. (4), the LIX values were obtained by considering the solid–aqueous phase distribution coefficient (*K*_d_), as described by Hall et al. (2015).

### Determination of human health hazards

The measurement of non-carcinogenic health hazards in adults and adolescents adhered to the established formulas set forth by the USEPA (2020). This assessment based on the pathway of herbicide ingestion from soil contaminated with herbicides, taking into account its implications for human health. To quantify the chronic daily intake (CDIi) of herbicide from non-dietary sources, specifically the inadvertent ingestion of contaminated soil by adults and adolescents, calculations were conducted employing the formula outlined by Bhandari et al. (2020), with results expressed in mg kg^−1^ day^−1^.

6 $$ {\text{CDI}}_{{\text{i}}} = \frac{{C_{s} \times EF \times ED \times IR_{i} }}{AT \times BW} \times CF $$

C_S_, representing the residual herbicide concentration in soil following a 50% degradation, is measured in mg kg^−1^. EF correspond to the frequency of exposure in days year^−1^, whereas ED indicates the duration of exposure in years. IR_i_ denotes the ingestion rate of herbicide contaminated soil in mg day^−1^. AT signifies the average lifespan in days, CF is the conversion factor, measured in kg mg^−1^ and BW represents the average body weight in kg (Table S1). Also, the values of projected non-dietary chronic daily intake (mg kg^‒1^ day^‒1^) that results from dermal contact with soil particles contaminated by herbicides, represented by CDI_d_ were obtained using the following equation of Bhandari et al. (2020).

7 $$ {\text{CDI}}_{{\text{d}}} = \frac{{C_{s} \times DA \times DAF \times AF \times EF \times ED}}{AT \times BW} \times CF $$

DA refers to the daily exposed dermal area (cm^2^ day^‒1^), with DAF representing the dermal adherence factor (mg cm^‒2^) specific to soil, and AF indicating the dermal absorption factor, as detailed in Table S1. The values of CDI_ih_, which denote the projected non-dietary daily intake (mg kg^−1^ day^‒1^) of herbicide-contaminated soil particles through the inhalation pathway, were measured using the formula (Bhandari et al., 2020):

8 $$ {\text{CDI}}_{{{\text{ih}}}} = \frac{{C_{{\text{s}}} \times EF \times ED \times IR_{ih} }}{PEF \times AT \times BW} $$

where, IR_ih_ denoting the inhalation rate (m^3^ day^‒1^) and PEF indicating the particle emission factor (m^3^ kg^‒1^) (Table S1).

The hazard quotient (HQ) represents the non-cancer risk associated with herbicides, and the hazard index (HI), which is the sum of individual herbicide HQ values, was computed following the equations outlined earlier (Afrin et al., 2021; USEPA, 2020):

9 $$ {\text{HQ}} = \frac{CDI}{{RfD}} $$

10 $$ {\text{HI}} = \sum HQ_{Herbicide} $$

where, the reference dose (RfD) serves as a benchmark for herbicide intake, measured in mg per kg per day. In adults and adolescents, the established maximum allowable reference oral doses (RfDs) for dimethenamid-P, metazachlor, and pyroxasulfone were 0.03, 0.20, and 0.02 mg per kg per day, respectively (APVMA, 2022).

### Statistical analysis

The experimental data were managed using Microsoft Excel (2016 edition). To establish the correlation (*P* < *0.05*) between different soil properties (predictors) and the DT_50_ values of herbicides in both agricultural and urban soils, multivariate analysis was performed using JMP Pro software. Principal component analysis (PCA) was employed to identify linear relationships (*P* < *0.05*) among environmental indices, soil *K*_d_, GUS, LIX, and DT_50_ values of dimethenamid-P, metazachlor, and pyroxasulfone. This analysis was conducted with Origin Pro Lab 2022 software.

## Results and discussion

### Properties of urban and agricultural soils

The pH values for the agricultural soils range from 6.78 (MAT) to 9.15 (QLD), while for the urban soils, the values range from 6.20 (CAL) to 6.60 (FLE). Electrical conductivity (EC) showed significant variation across all samples, ranging from 123.36 (CAL) to 190.46 (FLE) µS/cm. Total organic carbon (TOC) content ranged from 0.19% (QLD) to 2.02% (TAR) in agricultural soils, and from 1.29% (FLE) to 7.66% (CAL) in urban soils. Oxides of aluminum (Al) and iron (Fe) also varied, with the highest levels found in soil CAL (1.95% Al) and MAT (1.42% Fe), and the lowest in FLE (0.03% Al) and FLE (0.01% Fe). The selected soils belonged to four distinct textural classes such as loam, sandy loam, loamy sand, and silt loam, and the percentages of sand, silt, and clay ranged from 28.80 (QLD)–76.30 (MAT), 16.20 (MAT)–55.00 (TAR), and 7.40 (CAL)–30.00 (QLD), respectively. The major minerals detected and identified in the soils include quartz, albite, spinel, magnetite, oligoclase, hydrosodalite, sinnerite, bernalite, marshite, zeolite, and sodalite, with quartz being the most abundant. Various functional groups of soil OM detected in the soil samples were C=O, C–H, O–H, and C=C (Parven et al., 2024).

### Dissipation of herbicides in the tested soils

**Fig. 1:**
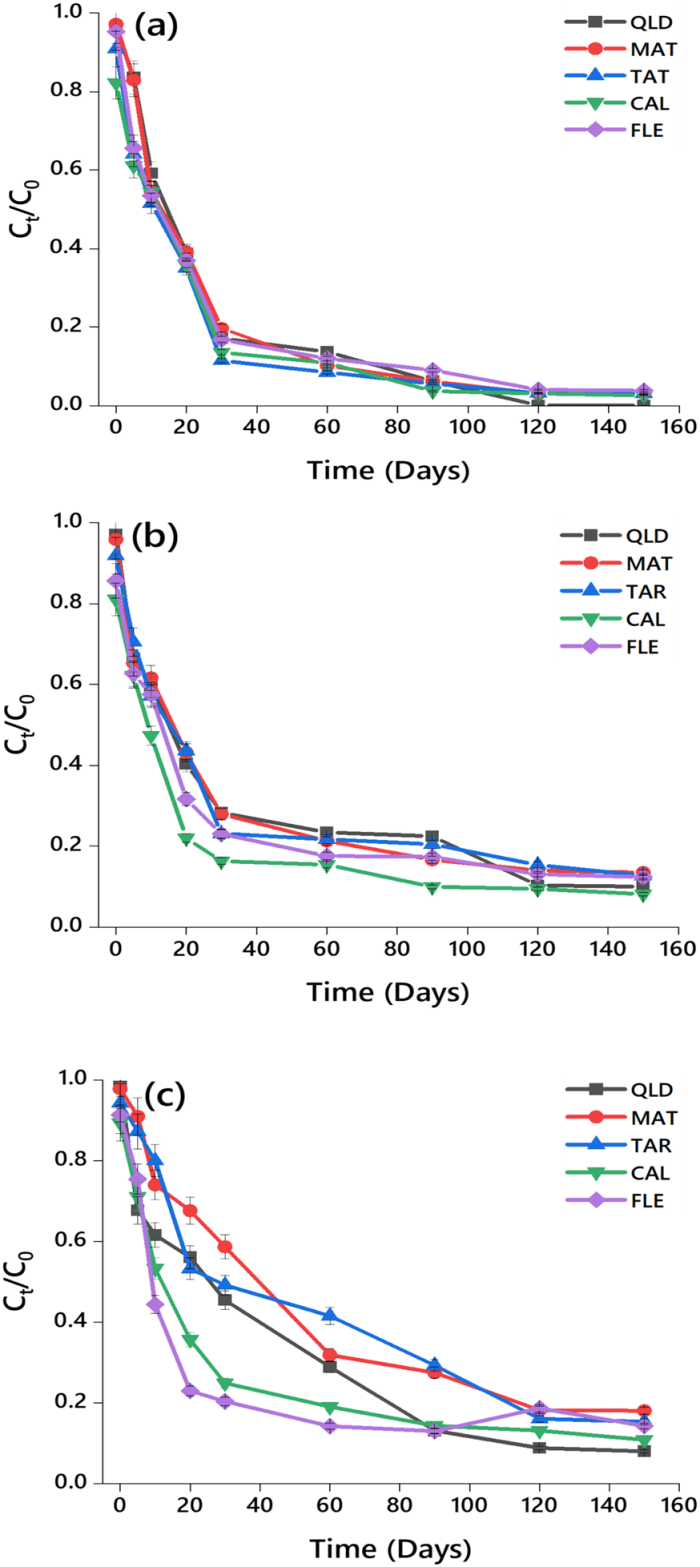
C_t_/C_0_ *versus* dissipation time of **a** dimethenamid-P, **b** metazachlor, and **c** pyroxasulfone in two urban soils (CAL and FLE) and three agricultural soils (QLD, MAT and TAR)

The *k* (day^‒1^) and DT_50_ (days) values of three herbicides for the urban and agricultural soils are shown in Fig. 1a–c and Table 1. The dissipation rate constant in soils for dimethenamid-P, metazachlor, and pyroxasulfone ranged from 0.010 (FLE) to 0.024 (MAT). The coefficient of determination (*R*^2^) values for these herbicides in soils were in the range of 0.87–0.93 for dimethenamid-P, 0.77–0.90 for metazachlor, and 0.56–0.97 for pyroxasulfone (Table S2). The calculated DT_50_ values, influenced by environmental factors, soil depth, and microbial activity (Oliveira et al., 2013), were between 29 and 35 for dimethenamid-P, 53 and 63 for metazachlor, and 43 and 69 for pyroxasulfone across the five tested soils (Table 1). Consequently, the pre-emergence herbicides demonstrated a trend for half-life in the chosen soils: pyroxasulfone > metazachlor > dimethenamid-P.

**Table 1:** Environmental and human health hazards of herbicide residues after 50% dissipation in two urban soils (CAL and FLE) and three agricultural soils (QLD, MAT, and TAR) based on experimental half-lives (DT_50_)

Herbicide	Soil ID	*k (*day^‒1^*)*	*K*_d_	DT_50_ (days)	GUS	LIX	Adults			Adolescent		
							HQ_i_	HQ_d_	HQ_ih_	HQ_i_	HQ_d_	HQ_ih_
Dimethenamid − P	QLD	0.020	1.62	35	3.47	0.33	5.23 × 10^‒5^	4.72 × 10^‒5^	2.71 × 10^‒5^	3.44 × 10^‒5^	6.73 × 10^‒9^	6.09 × 10^‒9^
	MAT	0.024	1.78	29	3.87	0.58	4.82 × 10^‒5^	4.35 × 10^‒5^	2.50 × 10^‒5^	3.17 × 10^‒5^	6.20 × 10^‒9^	5.61 × 10^‒9^
	TAR	0.022	1.76	32	4.43	0.78	4.55 × 10^‒5^	4.12 × 10^‒5^	2.36 × 10^‒5^	3.00 × 10^‒5^	5.86 × 10^‒9^	5.30 × 10^‒9^
	CAL	0.023	1.51	30	5.29	0.94	4.80 × 10^‒5^	4.34 × 10^‒5^	2.49 × 10^‒5^	3.16 × 10^‒5^	6.19 × 10^‒9^	5.59 × 10^‒9^
	FLE	0.020	1.78	35	4.43	0.76	4.72 × 10^‒5^	4.27 × 10^‒5^	2.45 × 10^‒5^	3.11 × 10^‒5^	6.08 × 10^‒9^	5.50 × 10^‒9^
Metazachlor	QLD	0.013	1.62	53	3.54	0.32	7.92 × 10^‒6^	7.16 × 10^‒6^	4.11 × 10^‒6^	5.21 × 10^‒6^	1.02 × 10^‒9^	9.22 × 10^‒10^
	MAT	0.012	1.56	58	3.63	0.35	8.17 × 10^‒6^	7.38 × 10^‒6^	4.24 × 10^‒6^	5.38 × 10^‒6^	1.05 × 10^‒9^	9.51 × 10^‒10^
	TAR	0.011	1.61	63	5.32	0.88	7.56 × 10^‒6^	6.84 × 10^‒6^	3.93 × 10^‒6^	4.98 × 10^‒6^	9.74 × 10^‒10^	8.81 × 10^‒10^
	CAL	0.013	1.71	53	6.30	0.97	6.27 × 10^‒6^	5.67 × 10^‒6^	3.26 × 10^‒6^	4.13 × 10^‒6^	8.08 × 10^‒10^	7.30 × 10^‒10^
	FLE	0.011	1.49	63	4.99	0.83	7.62 × 10^‒6^	6.89 × 10^‒6^	3.96 × 10^‒6^	5.02 × 10^‒6^	9.82 × 10^‒10^	8.87 × 10^‒10^
Pyroxasulfone	QLD	0.016	1.75	43	1.69	0.30	7.43 × 10^‒5^	6.72 × 10^‒5^	3.86 × 10^‒5^	4.89 × 10^‒5^	9.56 × 10^‒9^	8.65 × 10^‒9^
	MAT	0.012	1.66	58	2.07	0.23	7.77 × 10^‒5^	7.03 × 10^‒5^	4.03 × 10^‒5^	5.12 × 10^‒5^	1.00 × 10^‒8^	9.05 × 10^‒9^
	TAR	0.012	1.77	58	3.62	0.84	7.04 × 10^‒5^	6.37 × 10^‒5^	3.65 × 10^‒5^	4.64 × 10^‒5^	9.07 × 10^‒9^	8.20 × 10^‒9^
	CAL	0.012	1.67	58	4.69	0.97	7.06 × 10^‒5^	6.38 × 10^‒5^	3.66 × 10^‒5^	4.65 × 10^‒5^	9.09 × 10^‒9^	8.22 × 10^‒9^
	FLE	0.010	2.05	69	3.31	0.78	5.88 × 10^‒5^	5.32 × 10^‒5^	3.05 × 10^‒5^	3.87 × 10^‒5^	7.57 × 10^‒9^	6.85 × 10^‒9^

*k*, Dissipation rate constant; *K*_d_, calculated solid‒aqueous phase distribution coefficient; DT_50,_ Calculated half‒life; GUS, Groundwater ubiquity score; LIX, Leachability index; HQ, Hazard quotient via ingestion (HQ_i_), dermal (HQ_d_), and inhalation (HQ_ih_) pathways

These variations in dissipation rates are likely driven by factors such as soil texture, organic matter content, and microbial activity, which have been shown to significantly affect herbicide persistence (Oliveira et al., 2013). For instance, dimethenamid-P demonstrated the shortest half-life, with DT_50_ values ranging between 29 and 35 days across the five soils tested. This indicates a relatively rapid dissipation as compared to metazachlor and pyroxasulfone, which had DT_50_ values of 53–63 days and 43–69 days, respectively (Table 1). The increased persistence of pyroxasulfone suggests that it may pose a higher risk for carryover into subsequent planting seasons or nontarget contamination. This aligns with findings from previous studies that reported pyroxasulfone’s slower degradation rates in soils with lower microbial activity or higher clay content (Oliveira et al., 2013; Kaur et al., 2024). The observed hierarchy in herbicide half-life, i.e., pyroxasulfone > metazachlor > dimethenamid-P, highlights the importance of considering both chemical properties and site-specific factors in environmental risk assessments. The slower dissipation of pyroxasulfone, for example, could increase the potential for off-site movement via leaching or runoff, especially in areas with higher precipitation or irrigation. In contrast, dimethenamid-P, with its relatively faster degradation, may be a more suitable option in areas where minimizing residual activity is critical for crop rotation or environmental protection. Therefore, understanding the degradation dynamics of these herbicides under varying soil and environmental conditions is essential for optimizing their use in sustainable agricultural practices.

### Soil properties *versus* herbicide dissipation

The degradation of herbicides is significantly impacted by their accessibility to organisms and the physicochemical properties of the soil (Liu et al., 2021). Several herbicides attach strongly to soil particles and making them inaccessible for breakdown by organisms and causing them to persist in the soil for long periods (Curran, 2016). The rate at which herbicides including dimethenamid-P, metazachlor, and pyroxasulfone degraded in various agricultural and urban soils is directly linked to factors such as soil pH, %TOC, clay, silt, as well as the presence of iron and aluminium oxides, while it is inversely related to sand content. A detailed analysis, as depicted in Figure S1, confirms the correlation between these soil properties and the degradation rates of the herbicides. Among the factors examined, %TOC (*R*^2^ = 0.024), %clay (*R*^2^ = 0.063), %silt (*R*^2^ = 0.001), oxides of Fe (*R*^2^ = 0.189) and Al (*R*^2^ = 0.116), and soil pH (*R*^2^ = 0.084) showed a positive correlation with *k* (*R*^2^ = 1.0; *P* < 0.05) values of dimethenamid-P, metazachlor, and pyroxasulfone (Fig. S1). Conversely, the sand content exhibits a negative correlation (*P* < 0.05) with the degradation rates of these herbicides. These findings strongly indicate that most soil properties positively influence the dissipation rates of herbicides.

A trend of slower dissipation rates, indicated by higher DT_50_ values, was observed for various herbicides in different soils: FLE, QLD ˃ TAR˃ CAL˃ MAT and for dimethenamid-P, FLE, TAR ˃ MAT ˃ QLD, CAL for metazachlor, and FLE ˃ TAR, CAL, MAT˃ QLD for pyroxasulfone. Soils with higher OM or clay content generally showed reduced herbicide dissipation rates, leading to higher DT_50_ values (Table 1). The presence of both soil TOC and clay minerals significantly affected herbicide sorption, preventing microbial breakdown and thus prolonging their half-lives in both agricultural and urban soils. Surprisingly, urban soil CAL, despite containing the highest amount of mostly undecomposed OM at 7.66%, exhibited higher degradation rates, possibly due to its lower sorption capacity resulting shorter half-life. Therefore, the decomposed OM and clay content appears to be crucial in determining herbicide degradation rates in selected soils, as bound residues are less available for moving and degradation (Rasool et al., 2022). The minor differences in herbicide binding among the tested soils might be attributed to the existence of partly decomposed or undecomposed OM, detected floating in tubes and confirmed by SEM and SEM–EDS images (Parven et al., 2024). The chosen urban and agricultural soils displayed a wide range of TOC levels from 0.21 to 7.66%, and the detection of partially decomposed or undecomposed OM floating in tubes might explain the similar rates of herbicides degradation across the tested soils. Additionally, it is well recognised that herbicides strongly bind to well-decomposed OM, rendering them less available for biodegradation (Karasali & Pavlidis, 2020).

The robust binding of herbicides to TOC in both agricultural and urban soils is likely due to the existence of varied functional groups, including C=O, O–H, C–H, and C=C, which can form electrostatic, covalent, or hydrogen bonds. Particularly, the reactive nature of C=O and C–H groups influence various soil properties such as polarity, chemical reactivity, cation-exchange capacity, solubility, and wettability (Meftaul et al., 2021). Additionally, clay minerals, with their negatively charged surfaces composed of octahedral aluminate and tetrahedral silicate groups, can interact with herbicides through cation-exchange or electrostatic attraction mechanisms. Herbicide molecules bind to soil clay minerals by making surface complexes with metal ions (Wu et al., 2021). The dissipation of herbicides is notably hindered in soils with higher proportions of silt, oxides of Al and Fe as indicated by increased DT_50_ values. Soils such as TAR, CAL, FLE, and QLD, characterized by elevated silt content 55%, 41.20%, 23.80%, and 23.80%, respectively, exhibited slower herbicide degradation rates and prolonged DT_50_ values compared to MAT soil with 16.20% silt. Moreover, soils comprising higher levels of Al and Fe oxides in the clay fraction demonstrate reduced herbicide degradation rates, attributed to the strong affinity of negatively charged herbicide molecules for transition metals, forming complexes in soil solutions (Okada et al., 2020). Consequently, herbicide degradation tends to be slower in soils rich in TOC, clay, silt, and Al and Fe oxides, whereas TOC and clay playing crucial roles in this process.

The urban and agricultural soils analyzed in this study distributed with similar mineral compositions, with quartz being the dominant constituent, influencing herbicide degradation similarly across both types of soils. However, there were notable differences in urban and agricultural soils had slightly alkaline pH, and contained more sand, resulting in an accelerated degradation of herbicides. This acceleration can be attributed to the soils with slightly alkaline pH, which reduce the partitioning of herbicide molecules, likely due to electrostatic repulsion because of an abundance of net negative surface charges on soil clay minerals (Okada et al., 2020). Particularly, the rate of dissipation was slow down in soil CAL, with a pH of 5.80, while quicker dissipation occurred in soil QLD, with a pH of 9.15. Additionally, soil composition played a role, with soil MAT, consisting of 76.30% sand, exhibiting a higher herbicide degradation rate, whereas soil TAR and QLD, with 33.30% and 28.80% sand respectively, showed slower dissipation rates.

**Fig. 2:**
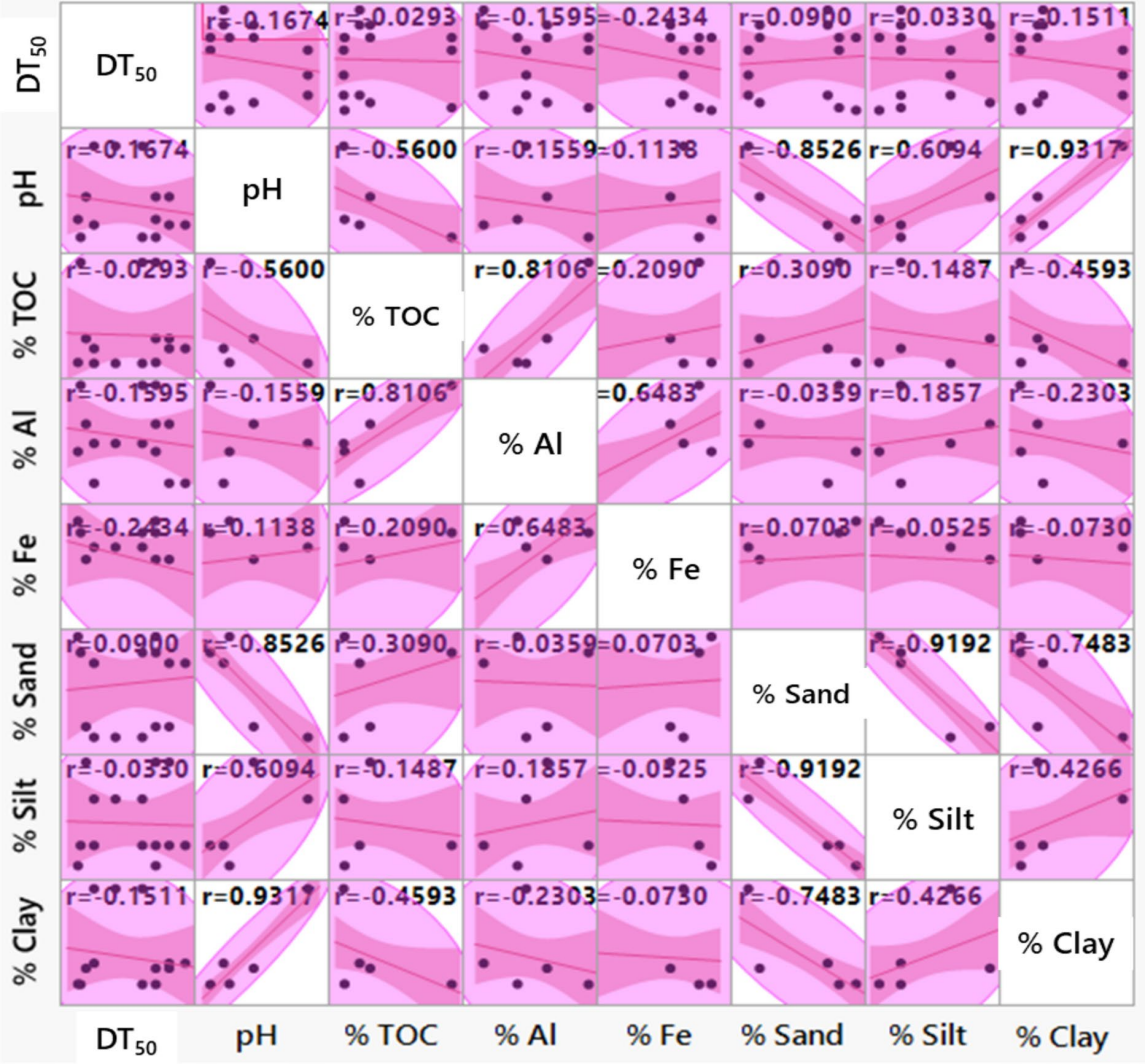
Relationship between soil properties (predictors), and half-life (DT_50_) values (outcome variable) of dimethenamid-P, metazachlor, and pyroxasulfone in urban (CAL and FLE) and agricultural soils (QLD, MAT and TAR)

Figure 2 presents the outcomes of multivariate analysis aimed at interpreting the relationships between soil characteristics (predictors) and DT_50_ values of herbicides. Among the predictors examined, TOC (*R*^2^ = – 0.029), %clay (*R*^2^ = – 0.151), %silt (*R*^2^ = – 0.033), Fe oxides (*R*^2^ = – 0.243), Al oxides (*R*^2^ = – 0.159), and soil pH (*R*^2^ = – 0.167) displayed significant negative correlations (*P* < 0.05) with DT_50_ values (*R*^2^ = 1.0) of dimethenamid-P, metazachlor, and pyroxasulfone. Conversely, sand content (*R*^2^ = 0.090) confirmed a positive correlation (*P* < 0.05) with DT_50_ of all selected herbicides (Fig. 2). These findings suggest that the dissipation of these pre-emergence herbicides in soils was influenced by various attributes, involving soil TOC, sand, clay, and silt content, as well as the presence of oxides of Al and Fe and soil pH. Nonetheless, in most instances, soil properties demonstrated a statistically negative correlation with the dissipation rate of dimethenamid-P, metazachlor, and pyroxasulfone.

The current findings related to DT_50_ values align with the reported half-life values for dimethenamid-P (Candia et al., 2012), metazachlor (Jursík et al., 2019), and pyroxasulfone (Mueller, 2017). The comparatively slower degradation rates and extended DT_50_ values observed only with pyroxasulfone and metazachlor could stem from their heightened affinity for soil OM, minerals, or dissolved organic carbon, thereby restricting the access of herbicide to microbial degradation (Peña et al., 2020). The decomposition of herbicides is likely to be influenced more by the amount and property of soil OM present (Marin-Benito et al., 2012). Nevertheless, some forms of OM can either hinder or enhance the rate of herbicide degradation by increasing sorption capacity (Liu et al., 2018), while a few of them promote degradation by influencing microbial activity (Kadian et al., 2008; Marin-Benito et al., 2012). Conversely, dimethenamid-P exhibits a rapid degradation rate, likely attributable to its decreased sorption capacity to the soil matrix (Sharipov et al., 2021). In general, herbicides are either bound to soil particles or dissolved in the aqueous phase upon entering the soil, and then desorbed and become available for microbial degradation (Kočárek et al., 2018). The comparatively weak or irreversible binding of herbicides to the soil aqueous phase, facilitating biodegradation, can be attributed to the presence of undecomposed or partially decomposed OM (Parven et al., 2024). In certain cases, herbicides that are bound to the dissolved organic carbon can become more bioavailable, leading to increased biodegradation (Marin-Benito et al., 2012).

### Soil DHA as a nontarget parameter of herbicide pollution

**Fig. 3:**
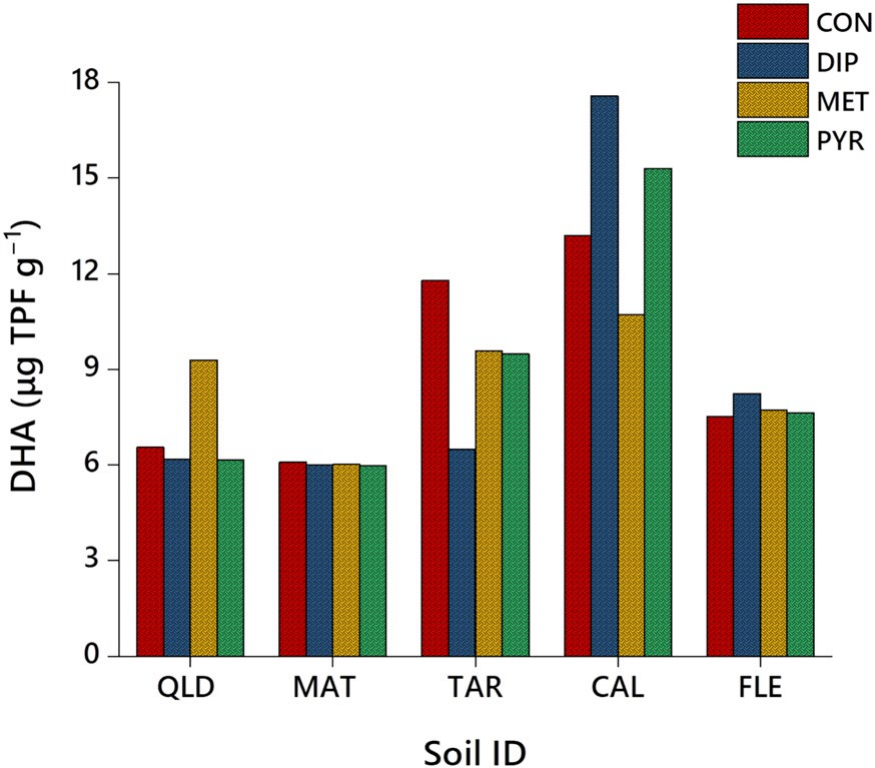
DHA (µg TPF g^–1^ soil) in urban soils (CAL and FLE) and agricultural soils (QLD, MAT and TAR), untreated and treated with dimethenamid-P (DIP), metazachlor (MET), and pyroxasulfone (PYR). CON = Control

DHA serves as a crucial indicator of soil health and microbial activity, frequently utilized to assess the impact of various hazardous chemicals, including herbicides, on soil microbiology (Wołejko et al., 2020). Figure 3 illustrates the effect of dimethenamid-P, metazachlor, and pyroxasulfone residues, after 50% degradation, on DHA in both agricultural and urban soils. DHA levels, measured in μg of TPF g^–1^ of soil, ranged from 6.09 (MAT) to 13.20 (CAL) for the control (untreated with herbicides), 5.99 (MAT) to 17.56 (CAL) for dimethenamid-P, 6.02 (MAT) to 10.72 (CAL) for metazachlor, and 5.97 (MAT) to 15.29 (CAL) for pyroxasulfone (Fig. 3). In control (untreated) agricultural soils, QLD, MAT, and TAR, the levels of DHA were notably higher compared to soils treated with the herbicides. Among the herbicide-treated agricultural soils with 50% residues of metazachlor after dissipation, activities of dehydrogenase were slightly higher than those with dimethenamid-P and pyroxasulfone (Fig. 3). In contrast, urban soils CAL and FLE, treated with dimethenamid-P, metazachlor, and pyroxasulfone exhibited increased levels of DHA compared to untreated soils. Among these urban soils, CAL displayed higher DHA levels, primarily due to its predominantly undecomposed organic matter, while FLE contained partially decomposed organic matter. Notably, among the herbicide-treated urban soils, residues of dimethenamid-P after 50% dissipation displayed higher DHA levels than metazachlor and pyroxasulfone. The correlations between soil DHA levels, both treated and untreated with dimethenamid-P, metazachlor, and pyroxasulfone, along with various urban and agricultural soil properties, were made through multivariate analysis (*P* < 0.05), and the data are presented in Figure S2. Among the array of soil parameters examined, significant positive correlations were observed between DHA values (*R*^2^ = 1.0; *P* < 0.05) of dimethenamid-P, metazachlor, and pyroxasulfone, and the %TOC (*R*^2^ = 0.868), Al oxides (*R*^2^ = 0.732), Fe oxides (*R*^2^ = 0.141), as well as sand (*R*^2^ = 0.131) and silt content (*R*^2^ = 0.018). Conversely, soil pH (*R*^2^ = – 0.406) and clay content (*R*^2^ = – 0.332) exhibited negative correlations (*P* < 0.05) with DHA values for all selected herbicides (Fig. S2). These findings underscore a predominantly positive relationship between most soil properties and herbicide DHA values except pH and %clay content.

The impact of herbicides on soil DHA is complex and varied. Depending on their type and concentration, herbicides can affect soil microbial communities, including those involved in DHA (Du et al., 2018). Some herbicides may hinder DHA by directly targeting key microbial populations, while others might indirectly enhance DHA by altering soil conditions or nutrient availability, thereby influencing microbial activity (Chettri et al., 2021; Łozowicka et al., 2021). Interestingly, urban soils exhibited significantly higher DHA levels compared to agricultural soils, probably because of the existence of partially decomposed or undecomposed OM, which stimulates microbial activity. Consequently, understanding the specific interactions between herbicides and soil microbial processes like DHA is essential for implementing sustainable agricultural practices and effective soil management strategies, especially with the introduction of new herbicides such as dimethenamid-P, metazachlor, and pyroxasulfone.

### Environmental health hazards of herbicide pollution

Concerns over herbicide contamination in waterbodies are escalating globally due to its adverse effects on nontarget biota, water quality, and food safety. Evaluating the potential for leaching is vital in calculating the environmental risks associated with herbicides, which hinges on *K*_OC_ values derived from *K*_d_. By applying DT_50_ and *K*_OC_ values, environmental indicators like GUS and LIX were computed, and the findings are summarized in Table 1. *K*_d_ values observed for dimethenamid-P, metazachlor, and pyroxasulfone across the selected soils ranged from 1.49 to 2.05 (Table 1), while *K*_OC_ values varied between 2.23 and 923 (Table S2).

The determined GUS values for dimethenamid-P, metazachlor, and pyroxasulfone ranged from 3.47 to 5.29, 3.54 to 6.30, and 1.69 to 4.69, respectively (Table 1). In general, soils are classified as leachers, transitional, or non-leachers based on GUS values > 2.80, 1.80–2.80, and < 1.80, respectively (Meftaul et al., 2023). Consequently, all the soils tested (QLD, MAT, TAR, CAL, FLE) are deemed potential leachers for dimethenamid-P and metazachlor, with QLD soil categorized as non-leacher, MAT soil as transitional, and TAR, CAL, and FLE soils as leachers for pyroxasulfone. Hence, the outcomes underscore the significant leaching potential of these herbicides in both agricultural and urban soils, potentially endangering water sources through contamination.

The determined LIX for dimethenamid-P, metazachlor, and pyroxasulfone ranged from 0.33 to 0.94, 0.32 to 0.97, and 0.23 to 0.97, respectively (Table 1). Usually, LIX fall between 0.0 and 1.0, denoting minimal to extensive leaching potential, as referenced by Martins et al. (2018) and Meftaul et al. (2023). The leaching potential of both agricultural and urban soils tested followed this sequence: CAL > TAR > FLE > MAT > QLD for dimethenamid-P and metazachlor, and CAL > TAR > FLE > QLD > MAT for pyroxasulfone. These results indicate a moderate to extreme leaching potential of the herbicides studied, potentially endangering both surface and groundwater reservoirs. Moreover, both GUS and LIX values affirm that these herbicides leach into water sources from the soil surface, demonstrating a potential hazard to water quality and nontarget biota.

To explore the combined effects of various environmental factors on the DT_50_ of herbicides, we conducted principal component analysis (PCA), the findings of which are illustrated in Figure S3. Notably, among the predictors examined, LIX (*R*^2^ = 0.097) *K*_oc_ (*R*^2^ = 0.081), and *K*_d_ (*R*^2^ = 0.074) demonstrated a significant positive correlation (*R*^2^ = 1.0, *P* < 0.05) with herbicide DT_50_, while the corresponding variance for the predictors and DT_50_ were 50.20, 22.80, 18.60, 6.90, and 1.50% respectively. The corresponding eigenvalues for the predictors were 2.51, 1.13, 0.93, 0.34 and 0.08, respectively (Fig. S3). The first two principal components accounted for around 50.2% of the cumulative variance, with an eigenvalue of 2.51. Conversely, GUS values exhibited a negative correlation (*R*^2^ = – 0.026, *P* < 0.05) with the DT_50_ of certain herbicides, namely dimethenamid-P, metazachlor, and pyroxasulfone.

### Human health hazards of herbicide pollution

**Fig. 4:**
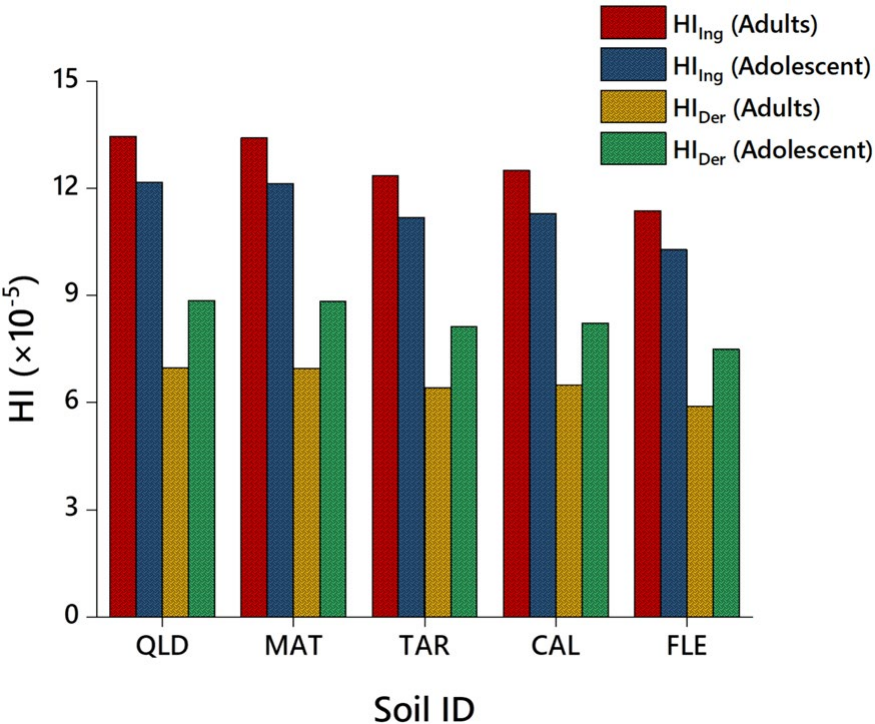
Potential non-cancer health hazards in terms of hazard index (HI) for human adults and adolescents exposed to urban soils (CAL and FLE) and agricultural soils (QLD, MAT and TAR), contaminated with dimethenamid-P, metazachlor, and pyroxasulfone residues after 50% dissipation

The potential health hazard of incidental exposure to herbicide-contaminated soil were assessed for adults and adolescents using established equations and parameters (Table S1). We utilized chronic daily intake (CDI) data to estimate the non-dietary exposure to herbicides via ingestion, dermal contact, and inhalation, employing well-recognized USEPA models. To assess potential non-cancer risks, we calculated the hazard quotient (HQ), comparing the estimated CDI to the reference dose (RfD) of the herbicide (Bhandari et al., 2020). Our findings on non-cancer risks associated with exposure to herbicide-contaminated agricultural and urban soils after 50% degradation is described in both Table 1 and Table S2. Notably, the computed CDI values (mg kg^‒1^ day^‒1^) for adults exceeded those for adolescents when exposing dimethenamid-P, metazachlor, and pyroxasulfone-contaminated soils via ingestion, dermal contact, and inhalation pathways (Table S2). Similarly, HQ values for adult exposure to certain herbicides were notably higher than those for adolescents across agricultural and urban soils (Table 1). Conversely, hazard index (HI) values for dimethenamid-P, metazachlor, and pyroxasulfone in these soils, obtained through adults, exposure via ingestion, dermal contact, and inhalation, were significantly higher than those for adolescents (Fig. 4 and Table S3). It’s worth noting that an HQ or HI value < 1 suggests a safe level of exposure to herbicides, while a value > 1 indicates a potential non-cancer health hazard (Bhandari et al., 2020; Parven et al., 2021). HQ and HI for three chosen herbicides across urban and agricultural soils were notably below the recommended threshold values. Our findings indicate that human exposure to herbicide residues, even after a 50% dissipation in urban and agricultural soils, poses minimal or no non-cancer risks via ingestion, dermal contact, and inhalation for adults and adolescents. Nonetheless, immediate exposure of pets and children to herbicide-treated ornamental plants, lawns, and parks in urban areas could pose potential health hazards, as described by Meftaul et al. (2021). This study represents the first comprehensive examination of potential hazards to human and environmental and soil health associated with the widespread use of dimethenamid-P, metazachlor, and pyroxasulfone in both agricultural and urban soils.

## Conclusion

We calculated the *k* and DT_50_ values of three pre-emergence herbicides, dimethenamid-P, metazachlor, and pyroxasulfone, involving agricultural and urban soils to gain insights into the potential threats of these hazardous chemicals to soil, human, and environmental health. This study revealed significant correlations between the *k* values (0.010–0.024 day^−1^) of the three herbicides and various soil properties. Specifically, a positive correlation was observed with TOC, clay, silt, and iron and aluminium oxides and pH, whereas a negative correlation was found with sand content. Conversely, the calculated DT_50_ values (29–69 days) of the three herbicides in urban soils displayed a negative correlation with TOC, clay, silt, soil pH, and iron and aluminium oxides, whereas a positive correlation was evident with sand content. From the soil health perspective, the increased DHA levels emphasized the role of organic matter in enhancing microbial activity. Furthermore, the environmental hazards, utilizing the GUS (1.69–6.30) and LIX (0.23–0.97) indices, indicated the potential of these herbicides in migrating from soil to waterbodies, posing risks to nontarget organisms. However, the evaluation of human non-cancer risks associated with herbicide exposure suggested minimal or non-existent risks after 50% degradation, based on HQ and HI < 1 indices calculated for adults and adolescents via ingestion, dermal contact, and inhalation pathways. To mitigate exposure risks and safeguard both the human and ecological health, it is advisable to employ improved formulations containing microbially-derived herbicides in urban and agricultural soils. Additionally, adopting precision band spraying techniques can help minimize herbicide usage, reduce transportation, and deter the development of resistant target organisms.

## Supplementary Information

Below is the link to the electronic supplementary material.

Supplementary file1 (PDF 453 KB)

## Data Availability

The datasets used in this study are available from the corresponding author on reasonable request.
