# Supplementary material for: Pre-emergence herbicides used in urban and agricultural settings: dissipation and ecological implications
Source: Environ Geochem Health. 2024 Nov 7;46(12):493. doi: 10.1007/s10653-024-02269-9 (PMC11543765; doi:10.1007/s10653-024-02269-9)
Supplement: Supplementary file 1 — Supplementary file1 (PDF 453 KB) [file 10653_2024_2269_MOESM1_ESM.pdf]

Supplementary Information

Pre-emergence herbicides used in urban and agricultural settings: dissipation and ecological implications

Aney Parven • Islam Md Meftaul • Kadiyala Venkateswarlu • Mallavarapu Megharaj*

A. Parven • I. M. Meftaul • M. Megharaj
Global Centre for Environmental Remediation (GCER), School of Environmental and Life Sciences, College of Engineering, Science and Environment, The University of Newcastle, Callaghan, NSW 2308, Australia

A. Parven • I. M. Meftaul
Department of Agricultural Chemistry, Sher-e-Bangla Agricultural University, Dhaka-1207, Bangladesh

K. Venkateswarlu
Formerly Department of Microbiology, Sri Krishnadevaraya University, Anantapuramu 515003, India

M. Megharaj
Cooperative Research Centre for Contamination Assessment and Remediation of the Environment (CRC CARE), The University of Newcastle, Callaghan, NSW 2308

****Address for correspondence***:
Prof. Mallavarapu Megharaj
Global Centre for Environmental Remediation (GCER)
College of Engineering, Science and Environment
The University of Newcastle, ATC Building
University Drive, Callaghan, NSW 2308, Australia
Mobile: +61 411126857; orcid.org/0000-0002-6230-518X

E-mail: megh.mallavarapu@newcastle.edu.au

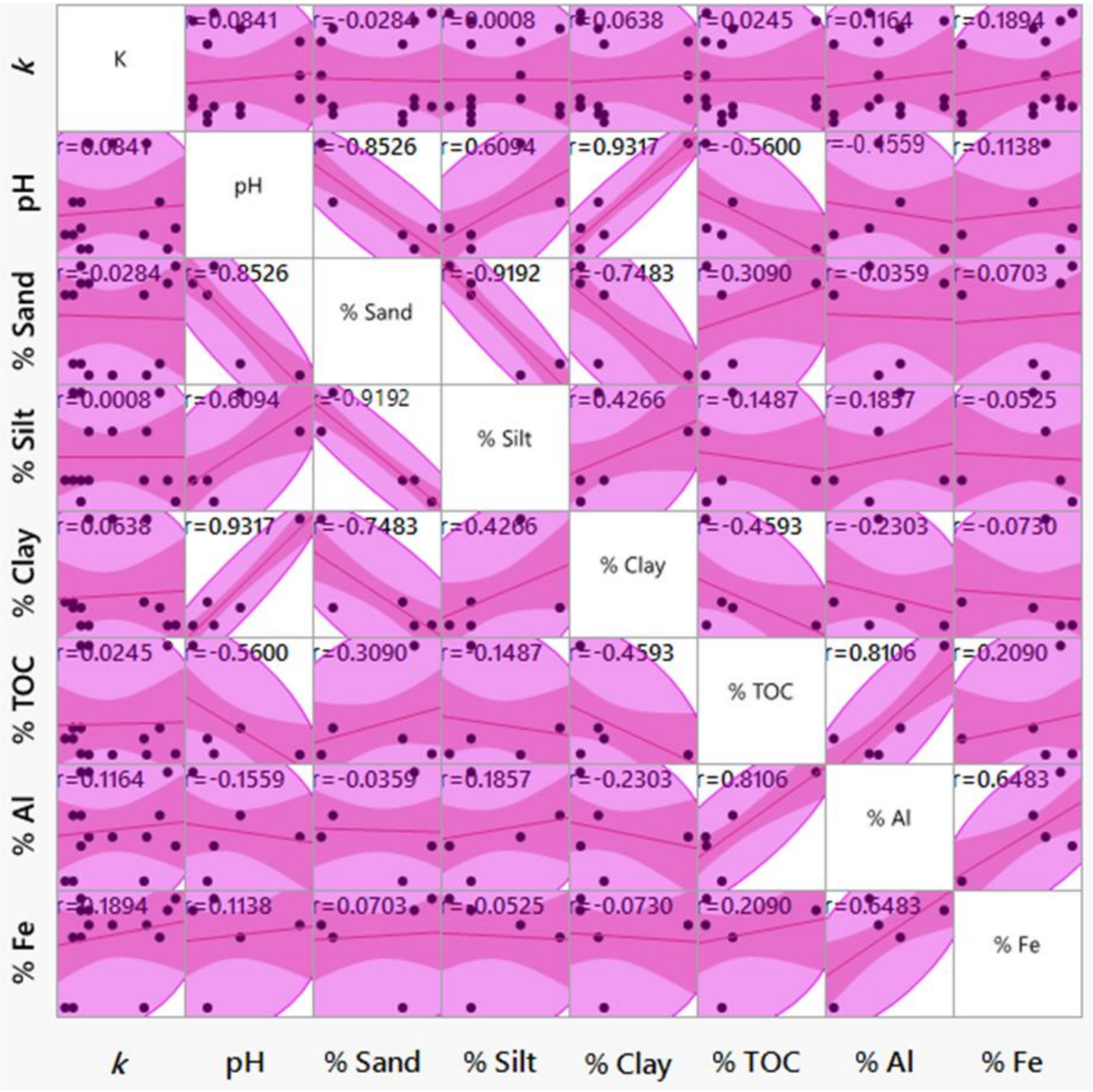

Fig. S1 Relationship between soil properties (predictors), and dissipation rate constant, *k* (day^{-1}) values (outcome variable) of dimethenamid-P, metazachlor, and pyroxasulfone in two urban soils (CAL and FLE) and three agricultural soils (QLD, MAT and TAR)

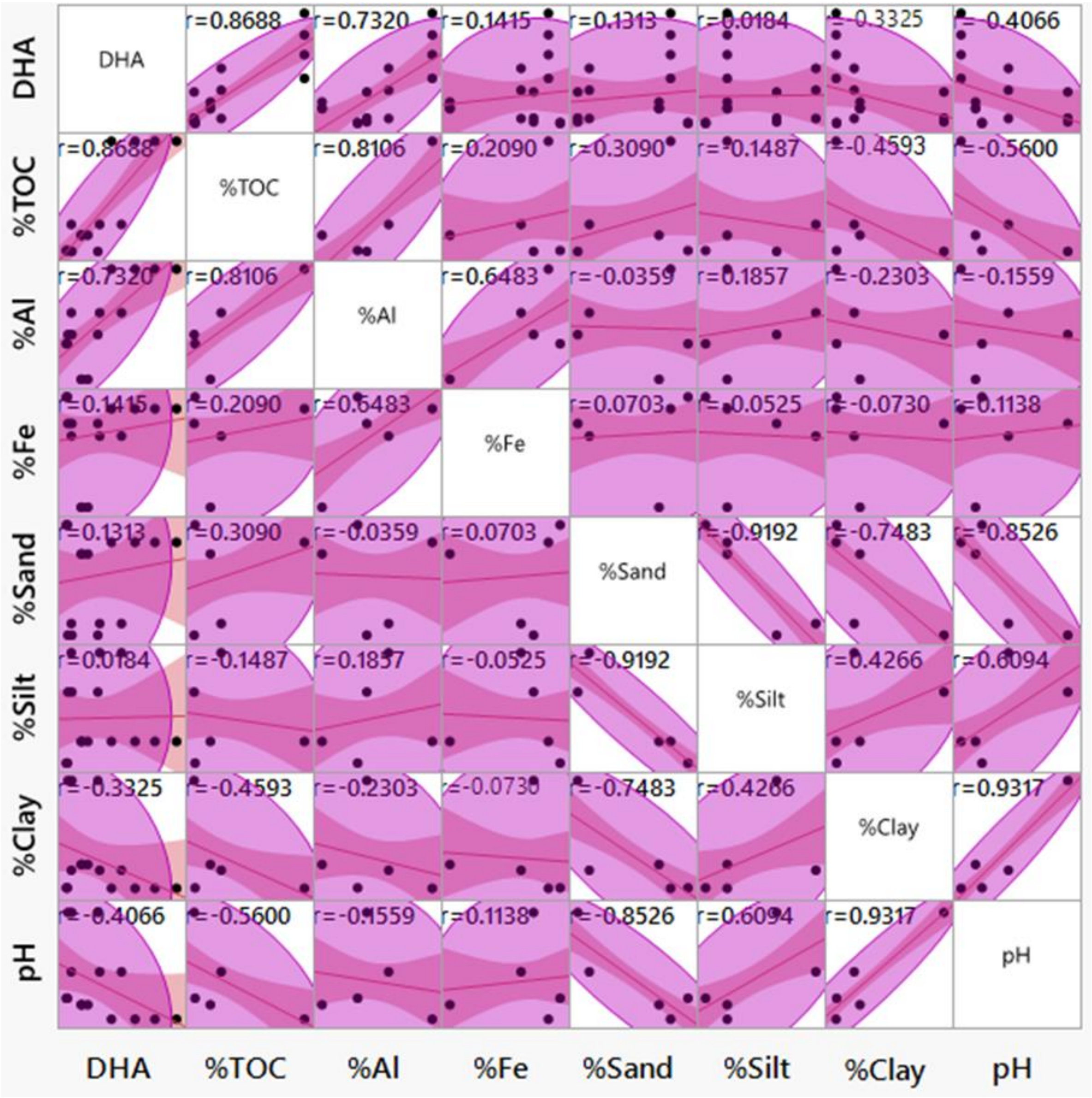

Fig. S2 Relationship between soil properties (predictors), and values of dehydrogenase activities (DHA, outcome variable) in two urban soils (CAL and FLE) and three agricultural soils (QLD, MAT and TAR) under the influence of dimethenamid-P, metazachlor, and pyroxasulfone

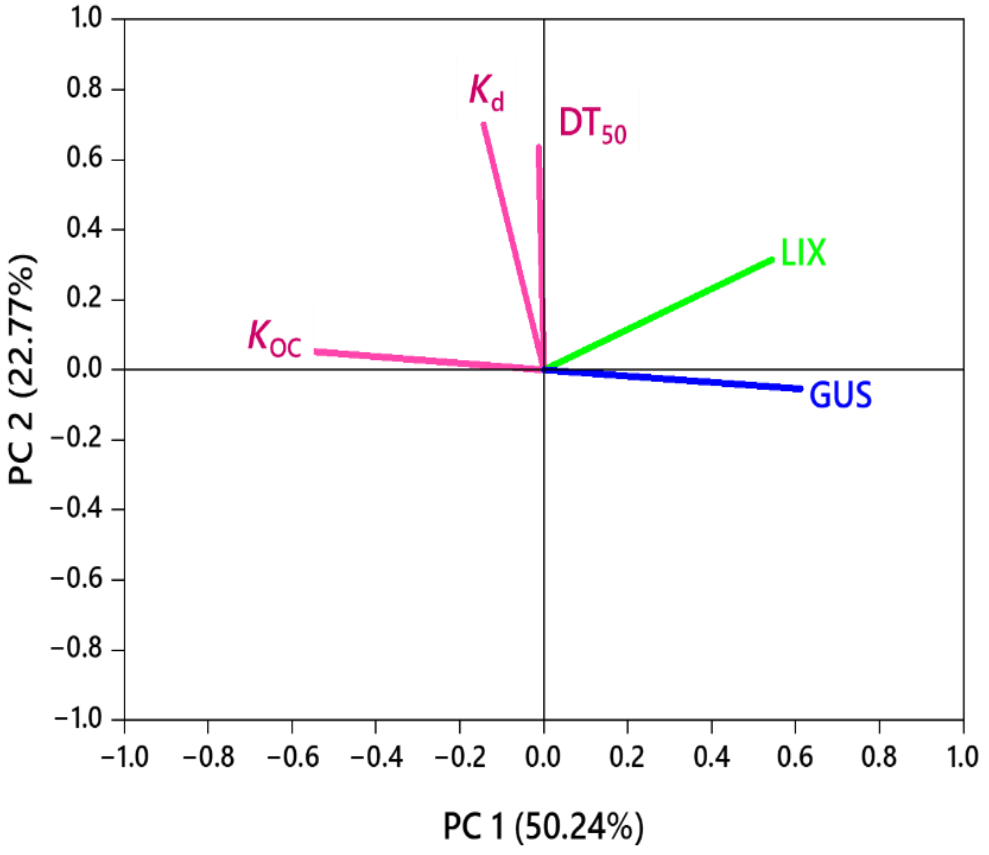

Fig. S3 PCA score plots showing relationships among the environmental parameters and half-life values (DT_{50}) of dimethenamid-P, metazachlor, and pyroxasulfone in two urban soils (CAL and FLE) and three agricultural soils (QLD, MAT and TAR)

Table S1 Constant parameters and their values for the estimation of non-cancer risk of the selected herbicides in humans (based on Bhandari et al. 2020; Meftaul et al. 2023)

Exposure factor	Adolescent	Adult
Ingestion rate (IR_{ing})	100 mg day^{-1}	100 mg day^{-1}
Body weight (BW)	32 kg	62 kg
Averaging lifetime (AT)	70 yrs (25,550 days)	70 yrs (25,550 days)
Exposed skin area (SA)	2,800 cm^2 day^{-1}	5,700 cm^2 day^{-1}
Exposure duration (ED)	14 yrs	30 yrs
Exposure frequency (EF)	350 days yr^{-1}	350 days yr^{-1}
Skin adherence factor (AF)	0.2 mg cm^{-2}	0.07 mg cm^{-2}
Skin absorption factor (SAF)	0.13 mg cm^{-2}	0.13 mg cm^{-2}
Conversion factor (CF)	1×10^{-6} kg mg^{-1}	1×10^{-6} kg mg^{-1}
Carcinogenicity slope factor (SF)	6.20×10^{-4} mg kg^{-1} day^{-1}	6.20×10^{-4} mg kg^{-1} day^{-1}
Inhalation rate (IR_{ih})	17.50 m^3 day^{-1}	17.50 m^3 day^{-1}
Particle emission factor (PEF)	1.36×10^9 m^3 kg^{-1}	1.36×10^9 m^3 kg^{-1}

Table S2 Concentration of herbicide residues in the soils after 50% dissipation **(**C_s), coefficient of organic carbon (K_{oc}), coefficient of determination (R^2) and estimated average non-dietary chronic daily intake (CDI), in terms of mg kg^{-1} day^{-1}, via ingestion (CDI_i), dermal (CDI_d), and inhalation (CDI_{ih}) pathways of herbicides for adults and adolescent in two urban soils (CAL and FLE) and three agricultural soils (QLD, MAT and TAR)

Herbicides	Soil ID	C_s (mg kg^{-1})	K_{OC} (L g^{-1})	R^2	Adults			Adolescent		
					CDI_i	CDI_d	CDI_{ih}	CDI_i	CDI_d	CDI_{ih}
Dimethenamid−P	QLD	2.36	55.33	0.92	$1.56×10^{-6}$	$8.13×10^{-7}$	$2.01×10^{-10}$	$1.41×10^{-6}$	$1.03×10^{-6}$	$1.83×10^{-10}$
	MAT	2.18	22.33	0.93	$1.44×10^{-6}$	$7.50×10^{-7}$	$1.86×10^{-10}$	$1.30×10^{-6}$	$9.52×10^{-7}$	$1.68×10^{-10}$
	TAR	2.06	11.02	0.87	$1.36×10^{-6}$	$7.09×10^{-7}$	$1.75×10^{-10}$	$1.23×10^{-6}$	$9.00×10^{-7}$	$1.59×10^{-10}$
	CAL	2.17	2.61	0.91	$1.44×10^{-6}$	$7.48×10^{-7}$	$1.85×10^{-10}$	$1.30×10^{-6}$	$9.49×10^{-7}$	$1.68×10^{-10}$
	FLE	2.13	13.14	0.91	$1.41×10^{-6}$	$7.35×10^{-7}$	$1.82×10^{-10}$	$1.28×10^{-6}$	$9.33×10^{-7}$	$1.65×10^{-10}$
Metazachlor	QLD	2.39	88.05	0.90	$1.58×10^{-6}$	$8.21×10^{-7}$	$2.03×10^{-10}$	$1.43×10^{-6}$	$1.04×10^{-6}$	$1.84×10^{-10}$
	MAT	2.46	87.37	0.84	$1.63×10^{-6}$	$8.47×10^{-7}$	$2.10×10^{-10}$	$1.47×10^{-6}$	$1.08×10^{-6}$	$1.90×10^{-10}$
	TAR	2.28	11.00	0.81	$1.51×10^{-6}$	$7.85×10^{-7}$	$1.94×10^{-10}$	$1.36×10^{-6}$	$9.96×10^{-7}$	$1.76×10^{-10}$
	CAL	1.89	2.23	0.77	$1.25×10^{-6}$	$6.51×10^{-7}$	$1.61×10^{-10}$	$1.13×10^{-6}$	$8.27×10^{-7}$	$1.46×10^{-10}$
	FLE	2.30	16.64	0.79	$1.52×10^{-6}$	$7.91×10^{-7}$	$1.96×10^{-10}$	$1.37×10^{-6}$	$1.00×10^{-6}$	$1.77×10^{-10}$
Pyroxasulfone	QLD	2.24	923.58	0.97	$1.48×10^{-6}$	$7.71×10^{-7}$	$1.91×10^{-10}$	$1.34×10^{-6}$	$9.78×10^{-7}$	$1.73×10^{-10}$
	MAT	2.34	666.85	0.95	$1.55×10^{-6}$	$8.06×10^{-7}$	$2.00×10^{-10}$	$1.40×10^{-6}$	$1.02×10^{-6}$	$1.81×10^{-10}$
	TAR	2.12	87.67	0.96	$1.40×10^{-6}$	$7.30×10^{-7}$	$1.81×10^{-10}$	$1.27×10^{-6}$	$9.28×10^{-7}$	$1.64×10^{-10}$
	CAL	2.13	21.89	0.84	$1.41×10^{-6}$	$7.32×10^{-7}$	$1.81×10^{-10}$	$1.27×10^{-6}$	$9.30×10^{-7}$	$1.64×10^{-10}$
	FLE	1.77	159.43	0.56	$1.17×10^{-6}$	$6.10×10^{-7}$	$1.51×10^{-10}$	$1.06×10^{-6}$	$7.75×10^{-7}$	$1.37×10^{-10}$

Table S3 Hazard index (HI) values for human adults and adolescent via ingestion (HI_i), dermal (HI_d), and inhalation (HI_{ih}) pathways based on the sum of HQ of individual herbicides in two urban soils (CAL and FLE) and three agricultural soils (QLD, MAT and TAR)

Soil ID	Adults			Adolescent		
	HI_{ing}	HI_{der}	HI_{inh}	HI_{ing}	HI_{der}	HI_{inh}
QLD	1.34×10^{-4}	1.21×10^{-4}	6.97×10^{-5}	8.86×10^{-5}	1.73×10^{-8}	1.57×10^{-8}
MAT	1.34×10^{-4}	1.21×10^{-4}	6.95×10^{-5}	8.83×10^{-5}	1.73×10^{-8}	1.56×10^{-8}
TAR	1.23×10^{-4}	1.11×10^{-4}	6.41×10^{-5}	8.14×10^{-5}	1.59×10^{-8}	1.44×10^{-8}
CAL	1.24×10^{-4}	1.13×10^{-4}	6.48×10^{-5}	8.23×10^{-5}	1.61×10^{-8}	1.45×10^{-8}
FLE	1.13×10^{-4}	1.02×10^{-4}	5.90×10^{-5}	7.49×10^{-5}	1.46×10^{-8}	1.32×10^{-8}

References

Bhandari, G., Atreya, K., Scheepers, P. T., Geissen, V. (2020). Concentration and distribution of pesticide residues in soil: Non-dietary human health risk assessment. *Chemosphere, 253*, 126594. https://doi.org/10.1016/j.chemosphere.2020.126594

Meftaul, I. M., Venkateswarlu, K., Annamalai, P., Parven, A., Megharaj, M. (2023). Degradation of four pesticides in five urban landscape soils: human and environmental health risk assessment. *Environmental Geochemistry and Health*, *45*(5), 1599–1614. https://doi.org/10.1007/s10653-022-01278-w
